# Diagnosing Diabetic Retinopathy in OCTA Images Based on Multilevel Information Fusion Using a Deep Learning Framework

**DOI:** 10.1155/2022/4316507

**Published:** 2022-08-04

**Authors:** Qiaoyu Li, Xiao-rong Zhu, Guangmin Sun, Lin Zhang, Meilong Zhu, Tian Tian, Chenyu Guo, Sarah Mazhar, Jin-Kui Yang, Yu Li

**Affiliations:** ^1^Faculty of Information Technology, Beijing University of Technology, Beijing 100124, China; ^2^Department of Endocrinology, Beijing Tongren Hospital, Capital Medical University, Beijing 100730, China; ^3^Beijing Diabetes Institute, Beijing 100730, China

## Abstract

**Objective:**

As an extension of optical coherence tomography (OCT), optical coherence tomographic angiography (OCTA) provides information on the blood flow status at the microlevel and is sensitive to changes in the fundus vessels. However, due to the distinct imaging mechanism of OCTA, existing models, which are primarily used for analyzing fundus images, do not work well on OCTA images. Effectively extracting and analyzing the information in OCTA images remains challenging. To this end, a deep learning framework that fuses multilevel information in OCTA images is proposed in this study. The effectiveness of the proposed model was demonstrated in the task of diabetic retinopathy (DR) classification.

**Method:**

First, a U-Net-based segmentation model was proposed to label the boundaries of large retinal vessels and the foveal avascular zone (FAZ) in OCTA images. Then, we designed an isolated concatenated block (ICB) structure to extract and fuse information from the original OCTA images and segmentation results at different fusion levels.

**Results:**

The experiments were conducted on 301 OCTA images. Of these images, 244 were labeled by ophthalmologists as normal images, and 57 were labeled as DR images. An accuracy of 93.1% and a mean intersection over union (mIOU) of 77.1% were achieved using the proposed large vessel and FAZ segmentation model. In the ablation experiment with 6-fold validation, the proposed deep learning framework that combines the proposed isolated and concatenated convolution process significantly improved the DR diagnosis accuracy. Moreover, inputting the merged images of the original OCTA images and segmentation results further improved the model performance. Finally, a DR diagnosis accuracy of 88.1% (95%CI ± 3.6%) and an area under the curve (AUC) of 0.92 were achieved using our proposed classification model, which significantly outperforms the state-of-the-art classification models. As a comparison, an accuracy of 83.7 (95%CI ± 1.5%) and AUC of 0.76 were obtained using EfficientNet. *Significance*. The visualization results show that the FAZ and the vascular region close to the FAZ provide more information for the model than the farther surrounding area. Furthermore, this study demonstrates that a clinically sophisticated designed deep learning model is not only able to effectively assist in the diagnosis but also help to locate new indicators for certain illnesses.

## 1. Introduction

Diabetic retinopathy (DR) is a microvascular impairment of the fundus caused by diabetes and is one of the leading causes of blindness and visual impairment [1]. DR has various and complex pathogenesis that are still unclear. For the diagnosis of DR, the most common methods are fluorescence fundus angiography (FFA) and indocyanine green angiography (ICGA) [2]. Both of these methods, however, are invasive medical imaging examinations. Moreover, the leakage of contrast media and retinal hemorrhage may disturb the media transparency, blurring the image of retinal vessels. As a result, the lesion area is difficult to precisely identify and assess.

Optical coherence tomography (OCT) is a new noninvasive imaging technique that can be used to effectively observe subtle changes in the superficial and deep capillary plexus of the human retinal microvasculature and has become popular in recent years [3]. As an extension of OCT, optical coherence tomographic angiography (OCTA) is used to capture and analyze the movement of blood cells in the field of vision by repeatedly capturing images of the same retinal position to obtain an image of the capillary network [4]. Studies have shown that several fundus diseases, such as age-related macular degeneration (AMD) [5], choroidal neovascularization (CNV) [6, 7], and retinal arterial macroaneurysms (RAM) [8], can be detected using OCTA images. OCTA is sensitive to the deterioration of vascular networks, hence providing a novel way of monitoring and evaluating the progression of DR [9, 10]. Liu et al. compared several machine learning models for DR discrimination based on 3 × 3 mm OCTA scans of different segmentation layers, including the superficial vascular plexus (SVP), deep vascular plexus (DVP), and retinal vascular network (RVN). The best DR diagnosis performance, with an overall accuracy of 0.82 and AUC of 0.83, was obtained by logistic regression regularized with the elastic net penalty (LR-EN) [11]. Abdelsalam and Zahran used a support vector machine (SVM) to diagnose early nonproliferative diabetic retinopathy (NPDR) based on multifractal geometry and obtained promising results [12]. However, due to the special imaging mechanism of OCTA, conventional image analysis technology does not always work well on OCTA images, and very different features are extracted. Moreover, the image quality of OCTA images is largely affected by factors such as a turbid refractive medium, image noise, and artifacts of vascular projection. Consequently, studies are urgently needed on feature extraction and analysis techniques for OCTA images.

Compared with traditional machine learning algorithms, deep learning shows higher performance in analyzing medical images [13–15]. The deep convolutional neural network (CNN) is one of the most common methods used to implement image segmentation and classification due to its powerful feature extraction and function fitting abilities [16–20]. Ma et al. published a dedicated Retinal OCTA SEgmentation (ROSE) dataset and proposed split-based coarse segmentation modules for vessel segmentation [21]. CNNs have also been considered for DR classification by jointly using en-face OCT and OCTA [22]. Currently, the number of OCTA data samples with high-quality labels is much smaller than that of fundus images; therefore, better utilization of multilevel information and a combination of domain knowledge is the key to improving deep learning-based OCTA analysis techniques.

In this paper, we proposed a deep learning framework that extracts and analyzes the multilevel information in OCTA images and demonstrated its advantage in DR diagnosis. We presented a segmentation model based on U-Net to segment the boundaries of vessels and the foveal avascular zone (FAZ) in OCTA images. Then, a new deep learning framework was proposed to predict the class of OCTA images based on fusing the original OCTA image and the segmentation results. A visualization method was used to indicate the regions of interest (ROIs) in the CNN model to locate the key lesion areas that are focused on by the prediction model to provide guidance for researchers on the key features for DR diagnosis.

## 2. Materials and Methods

### 2.1. Dataset

The OCTA-500 dataset compiled by Li et al. [23] was the dataset that was used in this study. Three different OCTA data projections were provided in this dataset. In this study, we used the maximum projection between ILM and OPL (B5), which is generated by the maximum projection of the inner retina and can clearly show the vascular morphology of the inner retina and the shape of the FAZ. Therefore, it is the most frequently used OCTA projection map for retinal vessel and FAZ segmentation [23].

**Figure 1:**
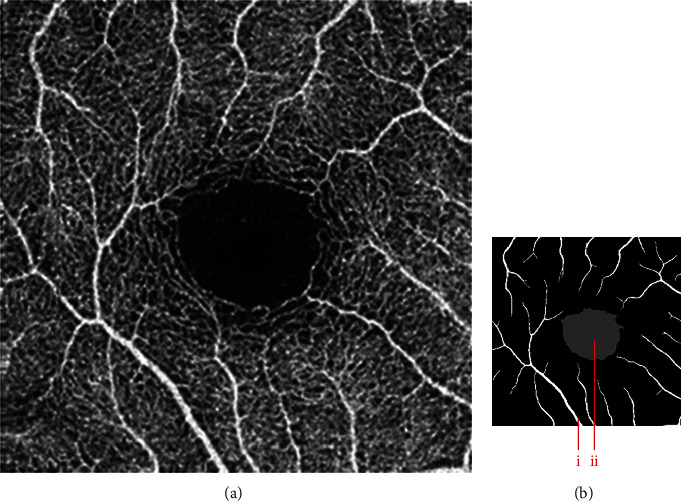
Examples of the OCTA image and the mask image. (a) OCTA image. (b) Mask image of (i) the main vessels (surrounding area) and (ii) the FAZ (center).

The dataset contains two subsets with different fields of view (FOVs). As shown in Figure 1(a), the white parts in the image are vessels, while the black zone in the center that is surrounded by vessels is the FAZ, which is a concave zone in the postretinal area that is approximately 2 mm in diameter and does not contain any vessels. One data subset had a 6 mm × 6 mm FOV, while the other subset had a 3 mm × 3 mm FOV. Fifty-seven DR samples and 244 normal samples were labeled in the dataset. The DR diagnosis was provided by ophthalmologists. Moreover, the masking labels of vessels and FAZ were provided.  Figure 1(b) shows one of the mask images: the white parts denote vessels, the gray part denotes the FAZ, and the black part is the background.

### 2.2. Segmentation of the Vessels and FAZ

**Figure 2:**
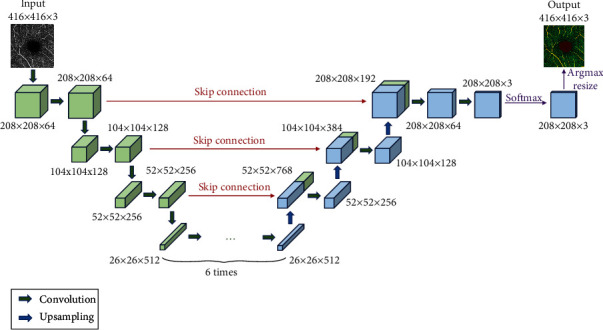
The segmentation model in this study based on the U-Net architecture.

To acquire the labeled image that indicates the vessels and FAZ, we proposed a segmentation method based on the U-Net [24] architecture, as shown in Figure 2. The OCTA image and its corresponding mask image are used as the input and ground truth, respectively. The network architecture can be divided into contracting and expansive paths. More specifically, the contracting path consists of a series of convolutional layers that reduce the size of the feature map. The expansive path is composed of upsampling operations and convolutional layers. The upsampling operations expand the size of the feature map, and the convolutional layers reduce the number of feature channels. The feature maps with the same size from contracting and expansive paths are concatenated by a skip connection. Eventually, the final segmentation result is given by the softmax and ArgMax operations.

The loss function is designed as follows:

(1) $$\begin{array}{c} L=-\frac{1}{H\times W}\left[\underset{m\in H}{\sum }\underset{n\in W}{\sum }y_{m,n}\log f_{m,n}\left(x\right)+\left(1-y_{m,n}\right)\log\left(1-f_{m,n}\left(x\right)\right)\right], \end{array}$$

where *H* denotes the height of the input image, *W* denotes the width, *y*_*m*,*n*_ denotes the true label of the sample, and *f*_*m*,*n*_(*x*) denotes the output of the segmentation model.

### 2.3. Image Channel Concatenation

**Figure 3:**
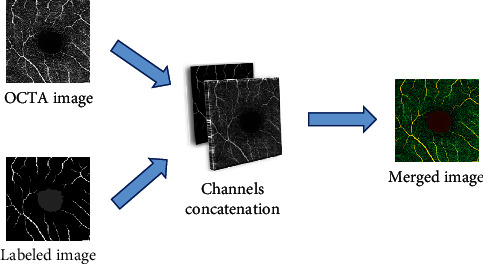
The process of obtaining a merged image with an OCTA image and a labeled image by channel concatenation.

The labeled images that indicate the vessels and FAZ can be used as expert opinions (i.e., domain knowledge), which provide more effective information to the deep learning model. Therefore, the labeled images are input with the OCTA images as additional domain knowledge. These two types of images are single-channel images. Therefore, they can be concatenated by the channel dimension to obtain double-channel images. In other words, the labeled image and OCTA image are two independent channels of the merged image. The concatenation process is shown in Figure 3.

### 2.4. Deep Learning Framework Based on Multilevel Information Fusion

**Figure 4:**
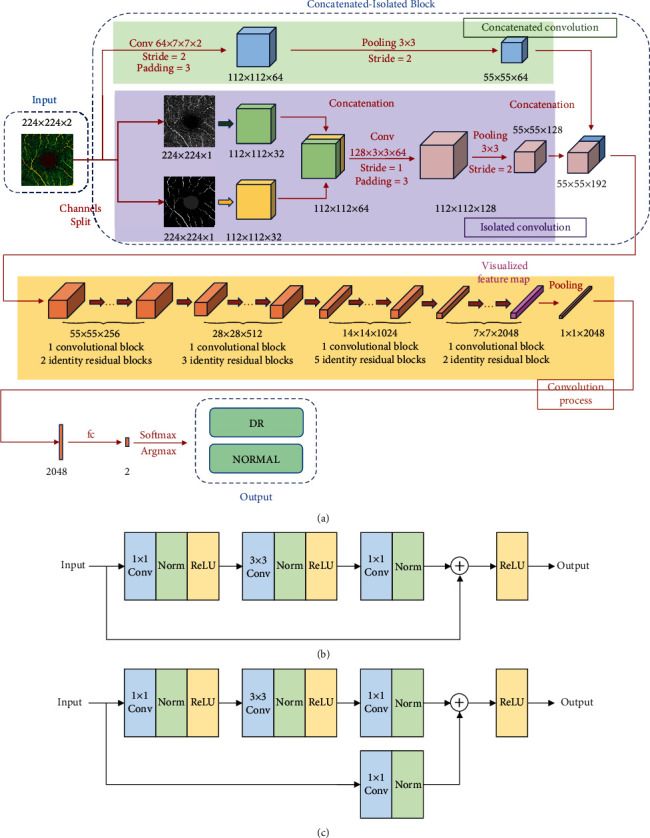
The CNN model used in this paper. (a) The total architecture, which consists of convolutional blocks and identity residual blocks in the convolution process. (b) The identity residual block. (c) The convolutional block.

The proposed deep learning framework for DR diagnosis in this study is designed based on the ResNet50 [25] architecture, and its structure is shown in Figure 4(a). To sufficiently extract information from the merged image, we designed a deep learning framework with an isolated concatenated block (ICB) architecture based on ResNet50. More specifically, in the isolated convolutional process, the input is a double-channel image composed of the original OCTA image and labeled image. They are separately processed by convolutional layers to extract the primary features. The two feature maps are concatenated to form a composite feature map. The new feature map is input into a convolutional layer and a pooling layer for information integration and parameter reduction. In the concatenated convolutional process, the input double-channel image is directly processed by a convolutional layer with a pooling layer to resize the feature map and make it consistent with the output of the isolated convolutional process. Eventually, the output feature maps of the isolated and concatenated convolutional processes are concatenated.

Then, the feature maps are processed by the following convolution process. More specifically, the convolution process is composed of four-stage residual convolutional blocks according to ResNet50. These blocks are made up of a convolutional block and several identity residual blocks. These block types are shown in Figures 4(b) and 4(c). Eventually, the classification results are given by a full connection layer followed by a softmax operation.

The loss function is designed as follows:

(2) $$\begin{array}{c} L=-\underset{c\in D}{\sum }t_{c}\log f_{c}\left(x\right), \end{array}$$

where *c* denotes the current class, *D* denotes all classes of the whole dataset, *t*_*c*_ denotes the true label of the sample, and *f*_*c*_(*x*) denotes the output of the classification model.

### 2.5. Model Visualization

In this study, we used gradient-weighted class activation mapping (Grad-CAM) [26] to visually analyze our model. Studies have shown that convolutional layers can retain spatial information [27, 28], while deeper layers contain more advanced feature information [29, 30]. Therefore, we focused on the last convolutional layer, which indicates the location of the region that is important for determining classification.

The class activation map *P*_*C*_ is calculated by the following algorithm:

(3) $$\begin{array}{c} P_{C}=\sigma \left(\underset{l}{\sum }\left(\frac{1}{N}\underset{i}{\sum }\underset{j}{\sum }\frac{\partial f_{C}}{\partial M_{ij}^{l}}\right)M^{l}\right), \end{array}$$

where *M*^*l*^ denotes the *l*th channel of the output feature map, *f*_*C*_ denotes the output of the sigmoid function of the current class, *N* denotes the number of feature points in the feature map, *σ* denotes the activation function, and a rectified linear unit (ReLU) is used in this study.

## 3. Results and Discussion

A total of 301 images were used for the training and testing process in the cross-validation. Among these images, the ophthalmologists labeled 57 images as DR and 244 as normal (the ground truth).

An accuracy of 93.1% and a mean intersection over union (mIOU) of 77.1% were achieved using our segmentation model. These values were calculated as the average of three classes. As shown in Table 1, the segmentation task includes three classes, namely, the background, vessels, and FAZ classes. The classification accuracy is 93.2% for the background, 93.8% for vessels, and 92.3% for the FAZ. Several typical segmentation results are shown in Table 2.

An ablation experiment was conducted to verify the performance of the proposed classification model. Three models were compared in the ablation test, namely, a model with only an isolated convolution process, with only a concatenated convolution process, and with both isolated and concatenated convolution processes. Obviously, the model using only the concatenated convolution process is equivalent to ResNet50, and the model using both isolated and concatenated convolution processes is the proposed model. Moreover, images with only segmentation results, only OCTA images, and merged images were taken as the input of the above three classification models. As shown in Table 3, the best accuracy of 88.1% with 95%CI ± 3.6% is achieved using the model using both isolated and concatenated convolution processes with merged images as the input. We also found that, given the same input, the model using both isolated and concatenated convolution processes achieved the best performance. While using the same model, the highest accuracy for each model was obtained using the merged images. Sixfold cross-validation was applied for the above analysis.

We compared our model with other existing models on the DR classification task for the same dataset with 6-fold cross-validation. In this comparison, the inputs for every model were merged images. As shown in Table 4, the highest accuracy of 88.1% with 95%CI ± 3.6% was achieved using our classification model. The sensitivity of our proposed model (51.8%) is also significantly larger than other methods, with a comparable specificity. Due to the unbalanced number of positive and negative training samples, the proposed model tends to underestimate the number of positive samples, which limits its sensitivity. However, this effect can be easily alleviated by setting another judging threshold to meet the need for a high sensitivity requirement of clinical diagnosis.

**Figure 5:**
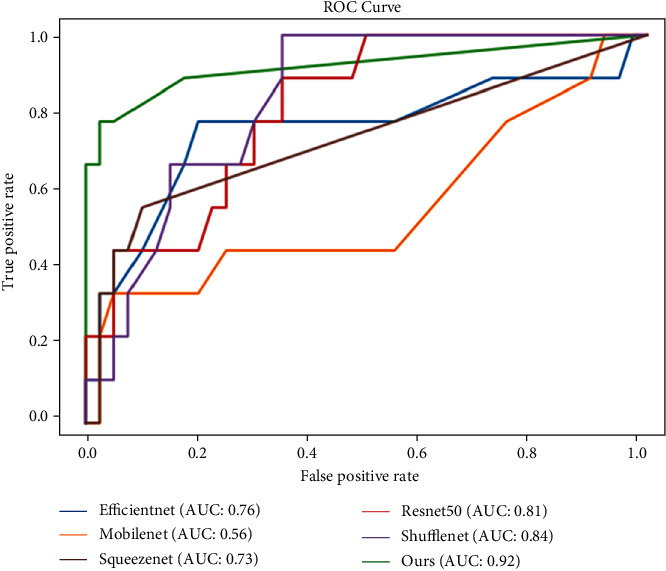
The ROC curves and AUCs of several CNN models.

The receiver operating characteristic (ROC) curves, which more comprehensively represent the performance of the classification model, of the above models are shown in Figure 5. The largest area under the curve (AUC) of 0.92 was obtained using our model.

**Figure 6:**
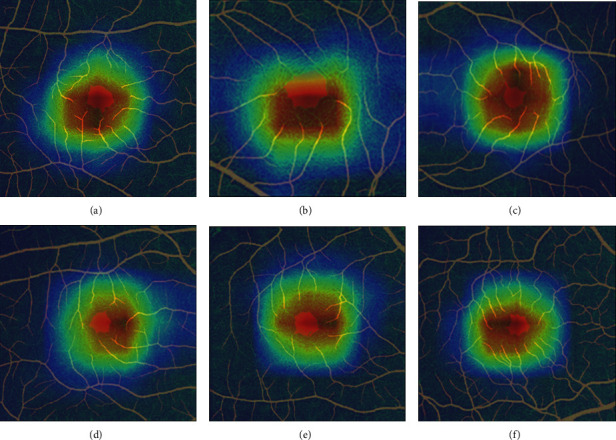
Examples of the visualization results.

The class activation maps of the last feature map were generated by Grad-CAM. The weight heatmaps were added to the merged images to indicate the ROIs for the classification model, as shown in Figure 6. Features in the region of the higher heatmap (in red) have a larger impact on the classification judgment. It can be observed that most red-colored regions are close to the central part of the images, which indicates that the FAZ and the vascular area around it are the most important regions considered by the model for DR classification. In other words, we found that pathological changes in DR may appear around the FAZ region based on the visualization results. In addition, compared to the narrow FOV images, the red color in the activation map has a smaller proportion of the whole image in the wide FOV image. We will explore more specific pathological changes through more experiments and by consulting medical experts in our future work.

We performed an experiment on another OCTA dataset, and the results are shown in Table 5. It was found that the highest accuracy was also achieved by using our model.

## 4. Conclusion

Deep learning can be used to analyze OCTA images by combining multilevel information and domain knowledge. The key discoveries in this study can be summarized as follows:

- The proposed deep learning framework with isolated and concatenated convolution processes significantly improved the accuracy of DR classification
- Fusing the information from the original OCTA image and labeled images that indicate the FAZ and vessel parts, which work as domain knowledge, provided more features and helped to lessen the DR classification errors
- Visualization analysis confirmed that the FAZ and the vascular region around it contain more useful information, such as the shape of the FAZ and the density of vessels around it, than the surrounding areas to distinguish DR samples from normal samples

The proposed analysis not only demonstrated the effectiveness of the deep learning algorithm and multilevel information fusion on DR diagnosis but also highlighted a potential indicator for DR in OCTA images. Hence, it was found that images with a larger FAZ area or a smaller density of vessels around the FAZ may be highly associated with the risk of DR in fundus screening. In the future, a study will be conducted on larger multicenter datasets, and the potential of the proposed deep learning framework in other related biomedical image analysis applications will also be explored.

## Figures and Tables

**Table 1:** The segmentation accuracy and IOU for every class using our model.

Class	Accuracy	IOU
Background	93.2%	92.6%
Vessels	93.8%	54.1%
FAZ	92.3%	84.6%
Average	93.1%	77.1%

**Table 2:** Examples of segmentation results.

Case	OCTA image	Label	Segmentation result
Case 1	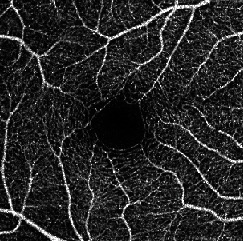	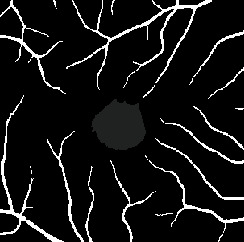	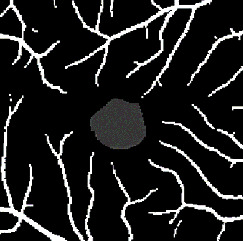
Case 2	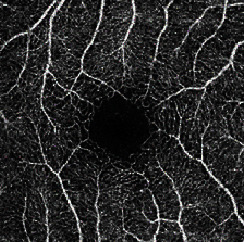	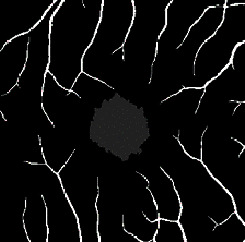	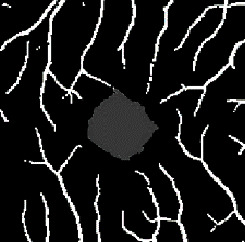
Case 3	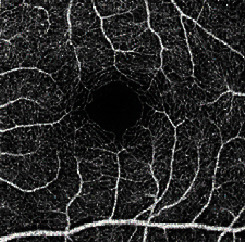	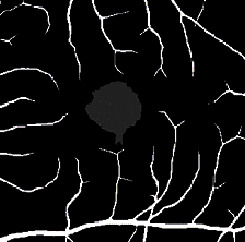	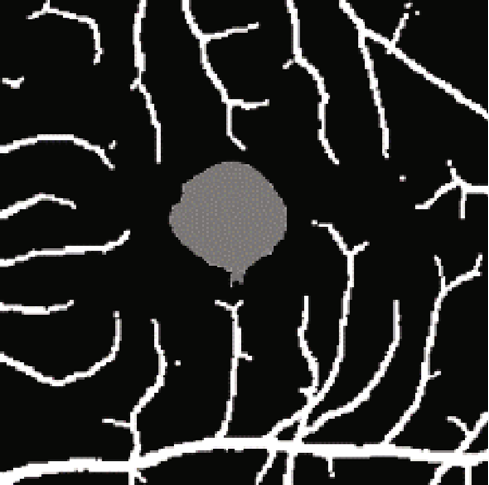

**Table 3:** The ablation experiment results of our classification model.

Input	Model	Accuracy
Segmentation results	Concatenated convolution (ResNet50)	75.2% (95%CI ± 7.5%)
	Isolated convolution	78.9% (95%CI ± 4.6%)
	Isolated and concatenated convolution	80.6% (95%CI ± 2%)

OCTA images	Concatenated convolution (ResNet50)	77.6% (95%CI ± 6.2%)
	Isolated convolution	84.4% (95%CI ± 2.7%)
	Isolated and concatenated convolution	87.8% (95%CI ± 3.1%)

Merged images	Concatenated convolution (ResNet50)	79.6% (95%CI ± 3.6%)
	Isolated convolution	84.7% (95%CI ± 2.0%)
	Isolated and concatenated convolution	88.1% (95%CI ± 3.6%)

**Table 4:** The classification results of several CNN models.

Model	Accuracy	Sensitivity	Specificity
EfficientNet	83.7% (95%CI ± 1.5%)	13.0% (95%CI ± 13.1%)	99.6% (95%CI ± 0.8%)
MobileNet v2	81.6% (95%CI ± 1.0%)	3.7% (95%CI ± 4.6%)	99.2% (95%CI ± 13.1%)
ResNet50	79.6% (95%CI ± 3.6%)	20.4% (95%CI ± 19.8%)	93.7% (95%CI ± 8.0%)
ShuffleNet v2	82.0% (95%CI ± 1.2%)	24.1% (95%CI ± 19.0%)	95.0% (95%CI ± 4.6%)
SqueezeNet	82.7% (95%CI ± 1.4%)	13.0% (95%CI ± 11.8%)	98.3% (95%CI ± 1.6%)
Ours	88.1% (95%CI ± 3.6%)	51.8% (95%CI ± 13.4%)	96.3% (95%CI ± 2.8%)

**Table 5:** The classification results on another OCTA dataset.

Model	Accuracy	Sensitivity	Specificity
EfficientNet	68.2% (95%CI ± 5.3%)	56.7% (95%CI ± 21.0%)	64.8% (95%CI ± 12.7%)
MobileNet v2	66.2% (95%CI ± 5.2%)	68.9% (95%CI ± 24.0%)	53.7% (95%CI ± 22.0%)
ResNet50	75.8% (95%CI ± 2.7%)	62.2% (95%CI ± 16.1%)	69.4% (95%CI ± 25.3%)
ShuffleNet v2	73.8% (95%CI ± 3.3%)	62.2% (95%CI ± 30.1%)	71.3% (95%CI ± 16.0%)
SqueezeNet	57.0% (95%CI ± 5.0%)	0.9% (95%CI ± 17.4%)	98.2% (95%CI ± 3.6%)
Ours	76.0% (95%CI ± 5.8%)	76.2% (95%CI ± 11.8%)	75.9% (95%CI ± 14.3%)

## Data Availability

The OCTA-500 dataset is publicly available at http://ieee-dataport.org/open-access/octa-500. The U-Net framework can be accessed through https://github.com/bubbliiiing/Semantic-Segmentation/tree/master/Unet_Mobile. The full code of the proposed method is available at https://github.com/liyuatbjut/OCTA-Analysis.

## References

[1] Kempen J. H., O’colmain B. J., Leske C., Haffner S. M., Friedman D. S. (2004). The prevalence of diabetic retinopathy among adults in the United States. *Archives of Ophthalmology*.

[2] Wang S., Zuo Y., Wang N., Tong B. (2017). Fundus fluorescence angiography in diagnosing diabetic retinopathy. *Pakistan Journal of Medical Sciences*.

[3] Akil H., Karst S., Heisler M., Etminan M., Navajas E., Maberley D. (2019). Application of optical coherence tomography angiography in diabetic retinopathy: a comprehensive review. *Canadian Journal of Ophthalmology*.

[4] Sambhav K., Grover S., Chalam K. V. (2017). The application of optical coherence tomography angiography in retinal diseases. *Survey of Ophthalmology*.

[5] Phasukkijwatana N., Tan A. S., Chen X., Freund K. B., Sarraf D. (2017). Optical coherence tomography angiography of type 3 neovascularisation in age-related macular degeneration after antiangiogenic therapy. *British Journal of Ophthalmology*.

[6] Jia Y., Bailey S. T., Wilson D. J. (2014). Quantitative optical coherence tomography angiography of choroidal neovascularization in age-related macular degeneration. *Ophthalmology*.

[7] Miere A., Oubraham H., Amoroso F. (2018). Optical coherence tomography angiography to distinguish changes of choroidal neovascularization after anti-VEGF therapy: monthly loading dose versus pro re nata regimen. *Journal of Ophthalmology*.

[8] Limia L. H., Cunha L. P. (2020). Angiographie par tomographie a coherence optique dans un cas de macroanevrismes arteriels retiniens multiples. *Journal Françaisd'Ophtalmologie*.

[9] Wang Y., Luo Y. (2018). The applications of optical coherence tomography angiography in diabetic retinopathy. *Annals of Eye Science*.

[10] Liu G., Xu D., Wang F. (2018). New insights into diabetic retinopathy by OCT angiography. *Diabetes Research and Clinical Practice*.

[11] Liu Z., Wang C., Cai X., Jiang H., Wang J. (2021). Discrimination of diabetic retinopathy from optical coherence tomography angiography images using machine learning methods. *IEEE Access*.

[12] Abdelsalam M. M., Zahran M. A. (2021). A novel approach of diabetic retinopathy early detection based on multifractal geometry analysis for OCTA macular images using support vector machine. *IEEE Access*.

[13] LeCun Y., Bengio Y., Hinton G. (2015). Deep learning. *Nature*.

[14] Ker J., Wang L., Rao J., Lim T. (2017). Deep learning applications in medical image analysis. *IEEE Access*.

[15] Chakrabarty N. A deep learning method for the detection of diabetic retinopathy.

[16] Ma N., Zhang X., Zheng H., Sun J. (2018). ShuffleNet v2: practical guidelines for efficient CNN architecture design. *Computer Vision – ECCV 2018*.

[17] Sandler M., Howard A., Zhu M., Zhmoginov A., Chen L. C. MobileNetV2: inverted residuals and linear bottlenecks.

[18] Iandola F. N., Han S., Moskewicz M. W., Ashraf K., Dally W. J., Keutzer K. (2016). SqueezeNet: AlexNet-level accuracy with 50x fewer parameters and <0.5MB model size. https://arxiv.org/abs/1602.07360.

[19] Tan M., Le Q. (2019). EfficientNet: rethinking model scaling for convolutional neural networks. https://arxiv.org/abs/1602.07360.

[20] Rawat W., Wang Z. (2017). Deep convolutional neural networks for image classification: a comprehensive review. *Neural Computation*.

[21] Ma Y., Hao H., Xie J. (2021). ROSE: a retinal OCT-angiography vessel segmentation dataset and new model. *IEEE Transactions on Medical Imaging*.

[22] Zang P., Gao L., Hormel T. T. (2021). DcardNet: diabetic retinopathy classification at multiple levels based on structural and angiographic optical coherence tomography. *IEEE Transactions on Biomedical Engineering*.

[23] Li M., Chen Y., Ji Z., Xie K., Li S. (2020). Image projection network: 3D to 2D image segmentation in OCTA images. *IEEE Transactions on Medical Imaging*.

[24] Ronneberger O., Fischer P., Brox T. U-Net: convolutional networks for biomedical image segmentation.

[25] He K., Zhang X., Ren S., Sun J. Deep residual learning for image recognition.

[26] Selvaraju R. R., Cogswell M., das A., Vedantam R., Parikh D., Batra D. (2020). Grad-CAM: visual explanations from deep networks via gradient-based localization. *International Journal of Computer Vision*.

[27] Zhou B., Khosla A., Lapedriza A., Oliva A., Torralba A. Learning deep features for discriminative localization.

[28] Xiong W., Du B., Zhang L., Hu R., Tao D. Regularizing deep convolutional neural networks with a structured decorrelation constraint.

[29] Khan A., Sohail A., Zahoora U., Qureshi A. S. (2020). A survey of the recent architectures of deep convolutional neural networks. *Artificial Intelligence Review*.

[30] Zeiler M., Fergus R. Visualizing and understanding convolutional networks.

